# Impacts of both reference population size and inclusion of a residual polygenic effect on the accuracy of genomic prediction

**DOI:** 10.1186/1297-9686-43-19

**Published:** 2011-05-17

**Authors:** Zengting Liu, Franz R Seefried, Friedrich Reinhardt, Stephan Rensing, Georg Thaller, Reinhard Reents

**Affiliations:** 1 vit w.V., Heideweg 1, 27283 Verden/Aller, Germany; 2 Christian-Albert-University, Institute of Animal Breeding and Husbandry, 24908 Kiel, Germany

## Abstract

**Background:**

The purpose of this work was to study the impact of both the size of genomic reference populations and the inclusion of a residual polygenic effect on dairy cattle genetic evaluations enhanced with genomic information.

**Methods:**

Direct genomic values were estimated for German Holstein cattle with a genomic BLUP model including a residual polygenic effect. A total of 17,429 genotyped Holstein bulls were evaluated using the phenotypes of 44 traits. The Interbull genomic validation test was implemented to investigate how the inclusion of a residual polygenic effect impacted genomic estimated breeding values.

**Results:**

As the number of reference bulls increased, both the variance of the estimates of single nucleotide polymorphism effects and the reliability of the direct genomic values of selection candidates increased. Fitting a residual polygenic effect in the model resulted in less biased genome-enhanced breeding values and decreased the correlation between direct genomic values and estimated breeding values of sires in the reference population.

**Conclusions:**

Genetic evaluation of dairy cattle enhanced with genomic information is highly effective in increasing reliability, as well as using large genomic reference populations. We found that fitting a residual polygenic effect reduced the bias in genome-enhanced breeding values, decreased the correlation between direct genomic values and sire's estimated breeding values and made genome-enhanced breeding values more consistent in mean and variance as is the case for pedigree-based estimated breeding values.

## Background

With the availability of the bovine genome sequence and the development of high-density arrays of single nucleotide polymorphism (SNP) markers, the accuracy of genetic predictions has improved compared to conventional breeding value estimations based on phenotypic data and pedigree [1-9]. In order to model genetic variation for quantitative traits, Meuwissen et al. [10] have proposed a genetic evaluation model that includes a large number of SNP markers simultaneously. This genomic model assumes that, all the loci that affect the trait are in linkage disequilibrium (LD) with at least one SNP marker and thus marker genotypes can be used as predictors for breeding values. A main advantage of the availability of genome-enhanced breeding values (GEBV) in dairy cattle comes from the improved accuracy in pre-selecting animals for breeding. Therefore, more and more countries have been implementing genomic evaluations in dairy cattle breeding. The genomic BLUP model, which has been used to include high-density SNP data in most of the dairy cattle applications [11-17], assumes that all SNP contribute equally to the genetic variance, because field data results support the infinitesimal model [11,15,18].

The reliability of genomic predictions strongly depends on the number of genotyped bulls in the reference population that is used to estimate SNP effects [15,18]. The increase in genomic reliability appears to be approximately linearly correlated with the number of reference bulls [15]. However, little is known on how the size of reference populations impacts the estimation of SNP effects. A German national genomic dataset has been used to study this question. Genomic models [10,15-17,19] usually assume that a given SNP marker chip, such as the Illumina Bovine54K (Illumina Inc., San Diego, CA), explains all the genetic variation of a trait, and as a consequence no residual polygenic effect (RPG) is typically fitted in genomic prediction [10,15-17,19]. Fitting the RPG effect can account for the fact that SNP markers may not explain all the genetic variance [13,20,21]. Including the RPG effect in the genomic model can also render the estimates of SNP effect less biased and more persistent over generations [22]. To investigate the impact of including an RPG effect on genomic prediction, a larger dataset from the EuroGenomics reference population [18] was used. The objectives of this study were to investigate (1) the impact of the size of a genomic reference population using German reference bulls on the estimation of SNP effects and on direct genomic values (DGV) and (2) the impact of including an RPG effect on the accuracy of genomic prediction using EuroGenomics reference bulls.

## Methods

### German national genomic and phenotypic data

Holstein bulls from the German national genomic reference population originating partially from the national genome project GenoTrack and partially from routinely genotyped populations, were genotyped using the Illumina Bovine50k (Illumina Inc., San Diego, CA). The genotyping was conducted after ethnical review and approval by the project committee. Only SNP with a minor allele frequency greater than 1% and a call rate threshold greater than 95% i.e. 45,181 SNP were used for the analysis. Since male animals have only one allele for the 533 markers on chromosome X, the procedure to estimate marker effects developed for markers with two alleles was modified for these SNP. A genotyped animal was excluded if less than 95% of all SNP markers were called. Deregressed EBV (DRP) and effective daughter contributions (EDC) were obtained from the January 2010 German national conventional evaluation for all bulls. Forty-four traits from seven trait groups were analysed: milk production (three traits), udder health (one trait), functional longevity (one trait), calving (four traits), female fertility (six traits), workability (four traits) and conformation (25 traits). Table 1 shows the number of genotyped bulls per year of birth in the analyzed reference and validation sets. A total of 10,487 animals were genotyped. The reference bull population for milk yield comprised 5,025 German Holstein bulls. To validate the genomic evaluation system, genotyped bulls born between September 2003 and December 2004 were used for validation, and 3,676 genotyped bulls born before September 2003 were used to estimate SNP effects. To compute DGV of validation bulls, the estimated SNP effects multiplied by genotype were summed, which were then combined with the conventional pedigree index from the reference population using the pseudo-record BLUP method [14,23] to derive GEBV. Subsequently, the combined GEBV of the validation bulls were compared with their actual deregressed EBV to validate the genomic model and to check the consistency of the genetic trend and variance based on GEBV versus EBV according to the Interbull genomic validation test procedure [24]. Realised reliabilities for the pedigree-based EBV and the combined GEBV of the validation bulls were computed as the square of observed correlations with deregressed EBV, adjusted for the average reliability of the conventional EBV of their daughters [18]. The gain in reliability from genomic information was calculated as the difference between the realised reliability of the pedigree-based EBV and the combined GEBV of the validation bulls.

**Table 1:** Genomic and phenotypic data^§ ^used for routine genomic evaluation and for the validation study in January 2010 for German Holstein bulls

Year of birth	Data for routine genomic evaluation		Data for genomic validation study	
	
	Nb of genotyped animals	Nb of bulls in reference population	Nb of bulls with daughters	Sum
			**Reference population**	

≤ 1997	621	614	614	
		
1998	411	404	404	
		
1999	473	458	458	
		
2000	558	518	518	3676
		
2001	562	509	504	
		
2002	618	509	507	
		
2003	1131	999	671	
			
			**Validation set**	
			
			328	1232
		
2004	1207	906	904	

2005	630	112		
		
2006-2009	4267			
		
Sum	10,487	5,025		4908

§ The trait milk yield is used as reference

### Scenarios to study the impact of the residual polygenic effect

To investigate the impact of including an RPG effect on GEBV, another dataset was used, which originated from the EuroGenomics collaboration [18]. This dataset comprised 17,429 genotyped Holstein bulls, representing 21.4 million daughters from the EuroGenomics countries i.e. France, Germany, Nordic countries and The Netherlands [18]. The total number of genotyped animals in the German Holstein population, including domestic candidates, was 26,191. Deregressed Multiple Across Country Evaluation (MACE) EBV from the April 2010 Interbull evaluation were used as dependent variables. In order to apply the Interbull genomic validation test [24], the genotyped bulls were divided into two groups: 14,494 reference bulls born before September 2003 and 1,377 German national validation bulls born between September 2003 and December 2004. The GEBV and parental average of pedigree-based EBV of the validation bulls were compared to their actual deregressed MACE EBV to evaluate the predictive ability of the genomic model. To investigate the impact of including an RPG effect on genomic predictions, three different percentages of residual polygenic variance to total genetic variance were considered, 5%, 10% and 15%. These three scenarios were compared to a scenario with a very small residual polygenic variance by setting the heritability of the RPG effect to 0.0001 [14], which was equivalent to 0.02% of the total genetic variance for milk yield. In order to determine the optimal residual polygenic variance for each trait in the German Holstein breed, a genomic validation study was conducted according to the Interbull genomic validation test [24], in which SNP effects were estimated using genotypic and phenotypic information of older bulls and the resulting GEBV of younger validation bulls were compared to their daughters' actual performance, i.e. deregressed EBV of the validation bulls. Observed regression coefficients of validation bulls' DRP on GEBV were compared to their expected value of 1. The scenario with observed regression coefficients close or equal to the expectation of 1 was chosen as the one with the most optimal residual polygenic variance.

In the literature [25,26], some concern has been raised that, under the BLUP genomic model, estimated SNP marker effects may model mainly family relationships. Solberg et al. [22] have suggested fitting an RPG effect to reduce this problem. In order to investigate whether incorporation of an RPG effect into the genomic model would reduce the correlation of animal DGV with EBV of sires in reference population, milk yield was analysed for the scenarios of residual polygenic variance of 0.02%, 5%, 10% and 20%.

### A genomic model for German Holstein cattle

The following BLUP SNP model was applied to the DRP of reference bulls:(1)

Where *q*_*i *_is the DRP of bull *i*, *μ *is a general mean, *ν*_*i *_is the RPG effect of bull *i*, *p *is the number of fitted SNP, *z*_*ij *_is a genotype indicator (-1 or 1 for the two homozygotes and 0 for the heterozygote) of marker *j *of bull *i*, *u*_*j *_is the random regression coefficient for marker *j*, and *e*_*i *_is the residual effect of bull *i*. The total additive genetic variance, , was obtained from a conventional pedigree-based analysis, e.g. for milk production traits [6] and for female fertility traits [7], and was partitioned into two components: the residual polygenic variance , where *w *is the proportion of additive genetic variance explained by the RPG effect, and additive genetic variance explained by the *p *markers . We assumed that all markers contribute equal genetic variance. The proportion of residual polygenic variance *w *was assumed to vary across traits. The optimal *w *value was determined by applying the Interbull genomic validation test [24]. Residual variance associated with the deregressed EBV *q*_*i *_was , where  is the error variance obtained from the pedigree-based evaluation and *φ*_*i *_is the EDC for bull *i*. The RPG was fitted in the same way as in conventional genetic evaluations, i.e. using full pedigree and the same grouping procedures of phantom parents [14].

Since the BLUP SNP model (1) has a large number of parameters, i.e. SNP effects that need to be estimated simultaneously, a Gauss-Seidel iteration with residual updating [27] was applied to estimate all the effects of model (1). To further improve convergence, the SNP were processed in descending order of heterozygosity.

## Results and discussion

### Genomic validation using German national data

Table 2 shows the results of genomic validation based on the national genomic and phenotypic data of German Holstein cattle. Gains in reliability were high in general, due to the large reference population, except for fertility and calving traits. For the three milk production traits, the gain in reliability was about 30%, with the highest gain found for fat yield. Low heritability traits, such as fertility traits and stillbirth, had the lowest gain in reliability, which can be partially explained by the fact that reliabilities of conventional EBV of the reference bulls were much lower than for other traits. The realised gains in reliability of conformation traits ranged between 10% and 28%.

**Table 2:** Realised reliabilities^§ ^of genomic EBV of German Holstein bulls using the German national reference population

Trait	Pedigree index	GEBV	Gain	Conformation	Pedigree index	GEBV	Gain
Milk yield	28	56	28	Stature	23	51	28
Fat yield	27	58	32	Angularity	24	47	23
Protein yield	32	59	28	Rump angle	28	52	24
Somatic cell score	33	59	26	Udder depth	22	48	26
Longevity	34	51	17	Udder support	27	45	18
NR56 heifer	18	25	7	Chest width	24	46	22
Days open	21	29	8	Rear leg set	15	31	16
Stillbirth maternal	18	27	9	Locomotion	14	24	10
Milking speed	28	57	25	Body condition score	18	38	20

^§ ^Realised reliability values are multiplied with 100

When the genomic reference population for German Holstein cattle was switched from the German national to the EuroGenomics reference population, the number of reference bulls increased from 5,025 to 17,429. Additionally, the dependent variable DRP was derived from MACE EBV, which included phenotypic information from foreign countries, in contrast to German national EBV. In comparison to the validation results from the German national reference population in Table 2, when the larger EuroGenomics reference population was used the gain in reliability over pedigree-based EBV was 12% greater on average across four of the analyzed traits, protein yield, somatic cell score, udder depth and non-return rate. A significant gain in genomic reliability has also been reported in another genomic validation study using the EuroGenomics reference population [18].

### Effect of the genomic reference population size

During the development of the German genomic evaluation system, a number of test runs were conducted over time, which enabled a comparison of the estimates of SNP effects across different reference populations. Table 3 shows the comparison among estimates of SNP effects for milk yield from eight genomic test runs, differing in the number of reference bulls. Because only a few young reference bulls added some daughter information over the time period of the test runs, the difference in phenotypic information on bulls already genotyped was neglected when interpreting the results in Table 3. As the number of reference bulls increased from 735 to 5,025, the observed variance of the SNP effect estimates increased more than five times. The estimate for the SNP with the largest effect increased continuously, up to 4.13 fold, as the size of the reference population increased. As expected, the correlation of SNP effect estimates was higher between any two runs, when the numbers of genotyped bulls were similar. Note that the correlation of SNP effect estimates is much lower than the correlation of DGV which was close to 1 for the reference bulls (unpublished data). It can be seen that even under the BLUP genomic model assuming equal variance for all markers, effect estimates can vary greatly between markers, and even more when new genotyped animals are added to the reference population.

**Table 3:** Impact of reference population size on the SNP effect estimates for milk yield

Phenotypic data of milk yield from conventional evaluations	Nb of reference bulls	Variance of SNP effect estimates^§^	Estimate of largest SNP effect^$^	Correlation of SNP effect estimates between evaluations						
				B	C	D	E	F	G	H
January 2009	735 (A)	1	1	0.81	0.56	0.50	0.46	0.43	0.41	0.41
April 2009	1088 (B)	1.49	1.46		0.69	0.61	0.55	0.53	0.50	0.50
	1939 (C)	2.61	2.45			0.83	0.72	0.69	0.65	0.65
	3081 (D)	3.71	3.10				0.86	0.84	0.79	0.78
August 2009	3684 (E)	4.38	3.63					0.95	0.88	0.87
	4339 (F)	4.78	3.90						0.92	0.92
January 2010	4896 (G)	5.12	4.10							0.98
February 2010	5025 (H)	5.22	4.13							

^§^Variance of SNP effect estimates of reference population A is set to 1; ^$^the largest (same) SNP effect estimate for the first reference population A is set to 1

Table 4 shows the correlations between DGV estimates from the most recent genomic evaluations (February 2010) with the largest reference population of 5,025 bulls and DGV from each of the previous test runs. For all selection candidates, born between 2006 and 2009 and for which no phenotypic information was available, correlations between DGV increased from 0.824 to 0.993 as the number of reference bulls increased from 1,939 to 4,896. Candidates with sires included in both reference populations had somewhat higher DGV correlations than those without a genotyped sire in the reference population; however this difference in DGV correlations almost disappeared when the number of reference bulls reached 4,896. When bulls changed from candidate to reference individuals from one run to the next, the correlations between their DGV were much lower, ranging from 0.72 to 0.875, as expected. The increase in DGV correlations due to the inclusion of more reference bulls clearly shows that the genomic prediction for candidates becomes more consistent with an increasingly larger reference population.

**Table 4:** Correlations of DGV of milk yield of genotyped German Holstein animals compared to the February 2010 genomic evaluation with 5025 reference bulls

Phenotypic data from conventional evaluation	Nb of reference bulls	Common reference bulls in this run and the February 2010 run	Reference bulls in the February 2010 run but not in this run	Common candidates in this run and the February 2010 run	Candidates with a sire in both reference populations?	
					yes	no
April 2009	1939	0.989	0.720	0.824	0.877	0.817
	3081	0.983	0.820	0.902	0.932	0.896
August 2009	3684	0.993	0.832	0.938	0.956	0.932
	4339	0.991	0.883	0.960	0.972	0.956
January 2010	4896	0.9996	0.875	0.993	0.997	0.991

### Impact of the residual polygenic effect

Estimated SNP effects from three scenarios using the EuroGenomics reference population were compared to the scenario with the lowest residual polygenic variance for milk yield (Table 5). The correlation of SNP effect estimates decreased only marginally with an increasing difference in residual polygenic variance assumed in the genomic model. Correlations were greater than 0.9, except for the correlation between the two most different scenarios with 0.02% and 20% residual polygenic variance (i.e. 0.86). As the residual polygenic variance increased, the variance of SNP effect estimates and the value of the estimate for the SNP with the largest effect decreased. Similar results were also obtained for all the other traits (data not shown).

**Table 5:** Impact of assumed variance of the residual polygenic effect on SNP effect estimates for milk yield based on the EuroGenomics reference population

Scenario regarding residual polygenic variance	Variance of SNP effect estimates^$^	Estimate of the largest SNP effect^†^	Correlation of SNP effect estimates between scenarios		
			A (5%)	B (10%)	C (20%)
M (0.02%)^!^	1	1	0.942	0.910	0.860
A (5%)	0.65	0.84		0.993	0.964
B (10%)	0.50	0.75			0.987
C (20%)	0.34	0.62			

^$ ^variance of SNP effect estimates of the scenario with the lowest residual polygenic variance (0.2%) was set to 1; ^† ^estimate of the largest SNP effect when the lowest residual polygenic variance (0.2%) was set to 1; ^! ^M: the scenario with the lowest residual polygenic variance assumes a residual polygenic heritability of 0.0001 which is equivalent to a 0.02% residual polygenic variance for milk yield

Table 6 shows the observed variance of estimated DGV defined as the sum of SNP marker effects and the variance of DGVt, which was defined as the sum of DGV and the estimate of the residual polygenic effect, and their correlations with conventional EBV for the reference bulls. It can be seen that the correlation between DGV and EBV decreased and the correlation between DGVt and EBV increased slightly with increasing residual polygenic variance. The variance of DGV estimates was also significantly lower for the scenarios with the higher residual polygenic variance. However, the observed variance of DGVt remained constant, indicating that the information lost from the DGV was captured by the residual polygenic effect for the reference bulls. For all scenarios, regressions of conventional EBV or DRP on DGV or RPG were unity for the reference bulls, and the regression intercepts were very close to zero (results not shown). The estimates of RPG effects and DGV were positively correlated for milk yield, with somewhat higher correlations for the scenarios with a higher percentage of residual polygenic variance, e.g. 0.42 and 0.47 for 5% and 20% residual polygenic variance respectively.

**Table 6:** Impact of the assumed variance of residual polygenic effects on DGV estimates for milk yield of reference bulls in the EuroGenomics reference population

Scenario regarding residual polygenic variance	Correlation of conventional EBV with		Variance of DGV/DGVt divided by variance of EBV	
	DGV	DGVt^$^	DGV	DGVt
M (0.02%)^!^	0.95	0.95	0.95	0.96
A (5%)	0.90	0.96	0.57	0.94
B (10%)	0.87	0.97	0.47	0.95
C (20%)	0.84	0.98	0.36	0.96

^$ ^DGVt represents the sum of the estimate based on SNP effects (DGV) and the residual polygenic effect estimate; ^!^M: the genomic model assumes a residual polygenic heritability of 0.0001 which is equivalent to a 0.02% residual polygenic variance for milk yield

Following the Interbull genomic validation test procedure [24], conventional deregressed EBV of the validation bulls were compared to their DGV or combined GEBV estimates, which were calculated based on the reduced subset of the reference population. Table 7 shows the correlations observed between deregressed EBV, without adjusting for the reliability contributed by the daughters' performance, and DGV or GEBV estimates for the validation bulls. These correlations were high, indicating a high reliability of the genomic evaluation with 14,494 reference bulls. The correlations between DGV and deregressed EBV decreased as the polygenic variance increased, especially for milk yield. In contrast, the correlations between GEBV and deregressed EBV decreased less when the polygenic variance increased or remained constant, e.g. around 0.72 for somatic cell score. Based on the relatively small decrease in correlations between DRP and DGV or GEBV, we can conclude that the impact of the assumed percentage of residual polygenic variance on accuracy is limited. Regression of conventional deregressed EBV of the validation bulls on their GEBV based on phenotypic information from previous generations can identify some possible biases of a genomic evaluation model [24]. The intercept of the linear regression model was not significantly different from zero for all traits. The estimate of the regression slope was nearly unity for the validation population according to the validation procedure [24]. A regression slope estimate that is lower (higher) than its expected value indicates that the variance of the GEBV is too high (too low). According to the regression slope estimates in Table 8, the optimal percentage of residual polygenic variance seems to vary across traits. For traits with a high heritability or reliability, e.g. production traits, somatic cell score, stature and rump angle, the optimal residual polygenic variance appeared to be less than 5%. For the conformation traits, rump width and body conditional score, 10% or higher residual polygenic variances gave the least biased GEBV estimates. Genomic validation results have revealed that either fitting a residual polygenic effect in the BLUP SNP model or blending the G matrix with the pedigree relationship matrix A in the G-matrix BLUP model [13,20,21] was necessary to avoid over-prediction of candidates' GEBV. The optimal proportion of genetic variance assigned to the RPG effect or the optimal weight on matrix A varies across traits. As a result, a trait-specific residual polygenic variance was assumed in routine genomic evaluations for German Holstein cattle. The magnitude of the assumed polygenic variance had a minor effect on the correlation between GEBV and deregressed EBV for selection candidates (Table 7); however, the variance of GEBV decreased significantly with increasing residual polygenic variance. Including the RPG effect in the genomic model (1) provided a similar scale of variances for GEBV and EBV, making them more comparable and consequently resulting in a more accurate joint ranking of genomic selection candidates and proven bulls. However, the problem of optimal partitioning of the additive genetic variance between the residual polygenic and SNP-based components is not resolved. More appropriate statistical methods, such as REML or Bayesian methods [28], should be used to estimate the residual polygenic variance, preferably also including non-genotyped animals.

**Table 7:** Pearson correlations of deregressed EBV with direct (DGV) or combined genomic value (GEBV) for the validation bulls using the EuroGenomics reference population

Trait	Correlation with DGV for scenarios with percent residual polygenic variance				Correlation with GEBV for scenarios with percent residual polygenic variance			
	
	M^§^	5%	10%	20%	M^§^	5%	10%	20%
Milk yield	0.76	0.73	0.71	0.70	0.76	0.75	0.74	0.74
Somatic cell score	0.72	0.71	0.70	0.68	0.72	0.73	0.72	0.72
Stature	0.73	0.73	0.72	0.70	0.72	0.71	0.71	0.71
Udder depth	0.72	0.71	0.70	0.68	0.70	0.70	0.69	0.68
Body conditional score	0.62	0.62	0.62	0.61	0.61	0.58	0.58	0.58

^§^M: the genomic model with the lowest residual polygenic variance assumes a residual polygenic heritability of 0.0001

**Table 8:** Estimates of the coefficient of regression of deregressed EBV on combined genomic value (GEBV) for the validation bulls using the EuroGenomics reference population

Trait	Scenarios for percent of residual polygenic variance			
	
	M^§^	5%	10%	20%
Milk yield	0.93	1.17	1.26	1.40
Fat yield	0.96	1.15	1.24	1.38
Protein yield	0.89	1.13	1.23	1.37
Somatic cell score	0.97	1.13	1.21	1.34
Longevity	0.97	0.83	0.90	1.00
Stature	0.91	1.00	1.09	1.21
Rump angle	0.96	1.05	1.12	1.22
Rump width	0.83	0.84	0.89	0.97
Udder depth	1.01	1.19	1.26	1.36
Body conditional score	0.95	0.94	1.00	1.09
Milking speed	1.01	1.06	1.11	1.19

^§^M: the genomic model with the lowest residual polygenic variance assumes a residual polygenic heritability of 0.0001

### Influence of the sires' EBV on direct genomic values

**Figure 1:**
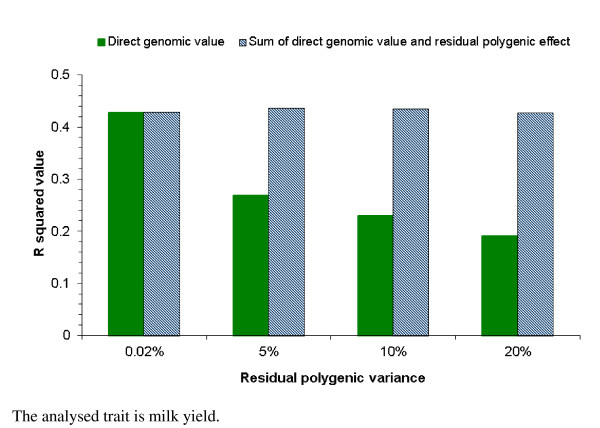
**Regression of direct genomic values of reference bulls on EBV of their sires with increasing residual polygenic variance**.

**Figure 2:**
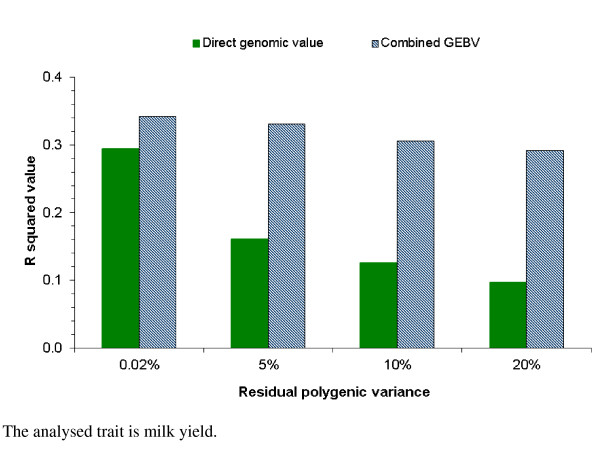
**Regression of direct genomic values of validation bulls on EBV of their sires with increasing residual polygenic variance**.

A concern that under the genomic BLUP model, animals' DGV are highly correlated with the sires' EBV [25,26] was addressed in this study by fitting an RPG effect with varying residual polygenic variances: 0.02%, 5%, 10% and 20% of the total genetic variance for milk yield. The DGV or the sum of DGV and RPG of 11,978 reference bulls that had genotyped sires in the reference population, were regressed on the conventional EBV of their 580 sires that were also included in the genomic reference population. The corresponding R^2 ^values indicate the fraction of the sons' genetic variation that is explained by their sires and are shown in Figure 1 for the genomic models with different residual polygenic variances for milk yield. When the RPG effect was given a nearly zero variance i.e. 0.02%, the R^2 ^value was 0.42 for both DGV and the sum. As the residual polygenic effect increased to 20% of the total genetic variance, the R^2 ^value between the DGV of the son and the EBV of the sire dropped below 0.20. In contrast to DGV, corresponding R^2 ^values for the sum of DGV and RPG remained constant, regardless of the level of residual polygenic variance. Figure 2 shows the influence of the sires' EBV on the DGV of validation bulls. The R^2 ^values of the regression of DGV on the sires' EBV dropped from 0.29 for the scenario with a 0.02% residual polygenic variance to about 0.10 for the scenario with a 20% residual polygenic variance, suggesting a decreasing impact of the sires' EBV on the DGV of validation bulls. With increasing residual polygenic variances, R^2 ^values decreased much less for combined GEBV of the validation bulls than for DGV alone, because in the combined GEBV the influence of sires was added back via the pedigree index. By fitting an RPG effect in the genomic model, the estimated DGV were less dependent on the sire's EBV, which was indicated by the lower R^2 ^value of the DGV regression on sire's EBV. The two figures showed that fitting an RPG effect in a genomic model can reduce the correlation between sires' EBV and animals' DGV.

### Estimation of SNP effects

Convergence of the BLUP SNP model was improved when the SNP markers were processed in descending order of heterozygosity. The processing order was particularly important when some reference bulls with extremely high or low EBV happened to have extremely high EDC, because those extreme phenotypic values could lead to extreme regression estimates of SNP markers with a low heterozygosity and thus could cause a convergence problem in the estimation of SNP effects. For the currently and most widely used 54 K Illumina BeadChip (Illumina Inc., San Diego, CA), we observed that SNP effects did not converge as well as their sum, i.e. DGV. Due to higher LD, convergence of SNP effects could become even lower for a higher density chip, although the convergence of DGV should remain unchanged. An alternative modelling of marker information from high-density chips should be explored.

## Conclusions

The tremendous advances in conventional genetic evaluations during the last decades have formed a solid basis for genomic evaluation and selection in dairy cattle. Genomic validation studies worldwide have demonstrated that the genomic model proposed by Meuwissen et al. [10] is highly effective to increase the reliability of evaluations in dairy cattle breeding. In this study, we have shown that the size of the genomic reference population is an important factor affecting the reliability of genomic prediction. Fitting a residual polygenic effect in the genomic model is necessary to avoid the variance of DGV being too high, to make the GEBV of candidates less biased, and to reduce the correlation between reference sires' EBV and animals' DGV. The optimal residual polygenic variance appears to differ between traits. Our validation study has clearly shown that genomic evaluation is efficient.

## Competing interests

The authors declare that they have no competing interests.

## Authors' contributions

ZL conducted the analyses and wrote the manuscript. FS prepared the genomic data. FR and SR helped check the results and suggested improvements. GT and RR coordinated the project, added valuable comments and suggestions. All authors read and approved the manuscript.

## References

[B1] Henderson CR Applications of Linear Models in Animal Breeding 1984 Guelph: University of Guelph Press

[B2] Quaas RL Computing the diagonal elements of a large numerator relationship matrix Biometrics 1976 32 949 953 10.2307/2529279

[B3] Schaeffer LR Kennedy BW Computing strategies for solving mixed model equations J Dairy Sci 1986 69 575 579 10.3168/jds.S0022-0302(86)80441-6

[B4] VanRaden PM Wiggans GR Derivation, calculation and use of national animal model information J Dairy Sci 1991 74 2737 2746 10.3168/jds.S0022-0302(91)78453-1 1918547

[B5] Schaeffer LR Dekkers JCM Random regression in animal models for test-day production in dairy cattle Proceedings of the 5th World Congress on Genetics Applied Livestock Production: 7-12 August 1994;Guelph 1994 443 446

[B6] Liu Z Reinhardt F Bünger A Reents R Derivation and calculation of approximated reliabilities and daughter yield-deviations of a random regression test-day model for genetic evaluation of dairy cattle J Dairy Sci 2004 87 1896 1907 10.3168/jds.S0022-0302(04)73348-2 15453507

[B7] Liu Z Jaitner J Reinhardt F Pasman E Rensing S Reents R Genetic evaluation of fertility traits of dairy cattle using a multiple-trait animal model J Dairy Sci 2008 91 4333 4343 10.3168/jds.2008-1029 18946139

[B8] Ducrocq V An improved model for the French genetic evaluation of dairy bulls on length of productive life of their daughters Anim Sci 2005 80 249 256

[B9] Schaeffer LR Multiple-country comparison of dairy sires J Dairy Sci 1994 77 2671 2678 10.3168/jds.S0022-0302(94)77209-X 7814738

[B10] Meuwissen THE Hayes BJ Goddard ME Prediction of total genetic value using genome-wide dense marker maps Genetics 2001 157 1819 1829 11290733 10.1093/genetics/157.4.1819 PMC1461589

[B11] Hayes BJ Bowman PJ Chamberlain AJ Goddard ME Invited review: Genomic selection in dairy cattle: Progress and challenges J Dairy Sci 2009 92 433 443 10.3168/jds.2008-1646 19164653

[B12] Loberg A Dürr JW Interbull survey on the use of genomic information Interbull Bull 2009 39 3 13

[B13] Van Doormaal BJ Kistemaker GJ Sullivan PG Sargolzaei M Schenkel FS Canadian implementation of genomic evaluations Interbull Bull 2009 40 214 218

[B14] Reinhardt F Liu Z Seefried F Thaller G Implementation of genomic evaluation in German Holsteins Interbull Bull 2009 40 219 226

[B15] VanRaden PM Van Tassell CP Wiggans GW Sonstegard TS Schnabel RD Taylor JF Schenkel F Invited review: Reliability of genomic predictions for North American Holstein bulls J Dairy Sci 2009 92 16 24 10.3168/jds.2008-1514 19109259

[B16] VanRaden PM Efficient methods to compute genomic predictions J Dairy Sci 2008 91 4414 4423 10.3168/jds.2007-0980 18946147

[B17] Daetwyler HD Pong-Wong R Villanueva B Woolliams JA The impact of genetic architecture on genome-wide evaluation methods Genetics 2010 185 1021 1031 10.1534/genetics.110.116855 20407128 PMC2907189

[B18] Lund MS de Roos APW de Vries AG Druet T Ducrocq V Fritz S Guillaume F Guldbrandtsen B Liu Z Reents R Schrooten C Seefried FR Su G Improving genomic prediction by EuroGenomics collaboration Proceedings of the 9th World Congress on Genetics Applied Livestock Production: 1-6 August; Leipzig 2010 150

[B19] Strandén I Garrick DJ Technical note: Derivation of equivalent computing algorithms for genomic predictions and reliabilities of animal merit J Dairy Sci 2009 92 2971 2975 10.3168/jds.2008-1929 19448030

[B20] Christensen OF Lund MS Genomic prediction when some animals are not genotyped Genet Sel Evol 2010 42 2 10.1186/1297-9686-42-2 20105297 PMC2834608

[B21] Aguilar I Misztal I Johnson DL Legarra A Tsuruta S Lawlor TJ Hot topic: A unified approach to utilise phenotypic, full pedigree, and genomic information for genetic evaluation of Holstein final score J Dairy Sci 2010 93 734 752 10.3168/jds.2009-2730 20105546

[B22] Solberg TR Sonesson AK Woolliams JA Ødegard J Meuwissen THE Persistence of accuracy of genome-wide breeding values over generations when including a polygenic effect Genet Sel Evol 2009 41 53 10.1186/1297-9686-41-53 20040081 PMC2813225

[B23] Ducrocq V Liu Z Combining genomic and classical information in national BLUP evaluations Interbull Bull 2009 40 172 177

[B24] Mäntysaari E Liu Z VanRaden PM Interbull validation test for genomic evaluations Interbull Bull 2010 41 10 14

[B25] Habier D Fernando RL Dekkers JCM The impact of genetic relationship information on genome-assisted breeding values Genetics 2007 177 2389 2397 18073436 10.1534/genetics.107.081190 PMC2219482

[B26] Habier D Tetens J Seefried FR Lichtner P Thaller G The impact of genetic relationship on genomic breeding values in German Holstein cattle Genet Sel Evol 2009 42 5 10.1186/1297-9686-42-5 PMC2838754 20170500

[B27] Legarra A Misztal I Technical note: Computing strategies in genome-wide selection J Dairy Sci 2008 91 360 366 10.3168/jds.2007-0403 18096959

[B28] Gianola D van Kaam BCHM Reproducing kernel Hilbert spaces regression methods for genomic assisted prediction of quantitative traits Genetics 2008 178 2289 2303 10.1534/genetics.107.084285 18430950 PMC2323816

